# Sexual and addictive risk behaviors and sexually transmitted infections in illegal gold miners in French Guiana: A multicenter observational study

**DOI:** 10.1371/journal.pone.0272932

**Published:** 2022-09-29

**Authors:** Louise Mutricy-Hureau, Amandine Pisoni, Martha Suarez-Mutis, Amanda Figueira da Silva, Yann Lambert, Pauline Mespoulhe, Audrey Godin, Marie-Claire Parriault, Astrid Van Melle, Emilie Mosnier, Mélanie Gaillet, Céline Michaud, Roxane Schaub, Muriel Galindo, Antoine Adenis, Mathieu Nacher, Stephen Vreden, Edouard Tuaillon, Maylis Douine

**Affiliations:** 1 Center for Clinical Investigation Antilles-Guyane, Inserm 1424, Cayenne Hospital, Cayenne, French Guiana, France; 2 UMR 1058 INSERM/EFS/University of Montpellier, Pathogenesis and Control of Chronic Infection, Montpellier University Hospital, Department of Bacteriology-Virology, Montpellier, France; 3 Oswaldo Cruz Foundation (Fiocruz), Laboratory of Parasitic Diseases, Rio de Janeiro, Brazil; 4 CRB Amazonie (Biological Resource Center), Cayenne Hospital, Cayenne, French Guiana, France; 5 Department of Infectious and Tropical Diseases, Cayenne Hospital, Cayenne, French Guiana, France; 6 Aix-Marseille University, INSERM, IRD, SESSTIM—Economic and Social Sciences, Health Systems, and Medical Informatics, F-13005, Marseille, France; 7 Centres Délocalisés de Prévention et de Soins (CDPS), Cayenne Hospital, Cayenne, French Guiana, France; 8 TBIP, University of French Guiana, University of Lille, CNRS, Inserm, Institut Pasteur de Lille, U1019-UMR9017-CIIL Center for Infection and Immunity of Lille, Lille, France; 9 Foundation for the Advancement of Scientific Research in Suriname (SWOS), Paramaribo, Suriname; Public Library of Science, UNITED STATES

## Abstract

**Objectives:**

Common representations of the world of gold mining–especially illegal–are usually negative: the activity conjures up images of drug trafficking, human exploitation, the sex trade, environmental destruction, and infectious diseases, in particular sexually transmitted infections (STIs). The aim of the present article is to describe the levels of risk behaviors such as transactional sex, multiple sexual partners, and the frequency of condom use, addictive substance consumption, and the prevalence of STIs among the population of illegal gold miners in French Guiana (FG), a French overseas entity in Amazonia, in order to guide potential interventions.

**Methods:**

An observational multicenter cross-sectional study was carried out from October to December 2019 along the two borders of FG with Suriname and Brazil at rest sites used by the miners.

**Results:**

Among the 499 participants, transactional sex was very prevalent, declared by 33.5% of men and 8.4% of women. Condoms were more frequently used for transactional sex than with a non-commercial partner (93.4% versus 42.1%). More women were tested for HIV than men (91.1% versus 55.2%). Excessive alcohol consumption (57.3%%) and tobacco use (41.2%) were very frequent, but cocaine or crack consumption was low (1.2%), which refuted our initial assumption. Consumers of alcohol had more sexual partners and reported condom use more frequently. Prevalence of HIV, HCV, HBV, and syphilis was respectively 0.5% (95% CI: 0.1–2.1), 2.1% (95% CI: 0.7–3.6), 1.6% (95% CI: 0.3–2.8), and 12.4% (95% CI: 9.0–15.7), which was higher than in the local population, especially for syphilis.

**Conclusion:**

This study documents for the first time the risk behaviors of gold miners in FG. Although the level of condom use was high, the prevalence of STIs combined with the high rate of transactional sex should encourage an increase in prevention and screening, in particular through rapid tests, given the mobility of the population concerned.

## Introduction

Common representations of the world of gold mining–especially illegal goldmining–are usually negative: the activity conjures up images of drug trafficking, human exploitation, the sex trade, adverse health effects (heavy metal poisoning, infectious diseases), and environmental destruction [1–3]. Migration, population movements, and remoteness from the health care system are unfavorable determinants of health linked to this activity. Sexually transmitted infections (STIs), in particular, are frequently associated with gold mining. This is mainly due to transactional sex, as described in sub-Saharan countries [4]. In 1998, at the biggest gold mining complex in the world in Carletonville, South Africa, 28.6% of mine workers, 68.6% of sex workers, 20.2% of men, and 37.1% of women in the community were infected with HIV [5]. In Mali, HIV prevalence in gold miners was eight times higher than the national prevalence (8.0% versus 1.3%) in 2015 [6].

In French Guiana (FG), about 10,000 illegal gold miners, mainly Brazilian (95%), work at more than 700 different mining sites located deep in the Amazon rainforest [2]. In 2015, a cross-sectional study revealed poor health among this population, including a high prevalence of malaria [7], high blood pressure, and leishmaniasis [2]. Assessment of STIs in this study showed a higher prevalence of HIV, hepatitis B (HBV), and syphilis than in other at-risk populations in FG, but with wide confidence intervals due to the sample size calculated to study malaria [8]. People from Brazil seem to be more affected by HIV: they represent 78% of newly-diagnosed patients seen in primary care centers [9], and their infection is diagnosed at a more advanced stage than that of French patients [10]. Gold mining often goes hand in hand with transactional sex [11,12]. Distinguishing between our representations and facts requires data that are difficult to obtain because these highly stereotyped populations are hard to reach and to study. Thus, documenting the situation is important to enable adequate public health strategies to be designed. Despite the high risk of transmission, very little is known about STIs at gold mining sites. In the 2015 study among gold miners, 9.3% of women declared sex work activity [2]. In addition, 65% of female sex workers (FSW) in Oiapoque (a town on the border between FG and Brazil) reported having previously worked at a gold mine [12].

In this context, we hypothesized that behaviors leading to an increased risk of STIs are more prevalent in the population of illegal gold miners in French Guiana.

Five years after the first cross-sectional study, a second study was carried out to evaluate an intervention to control malaria (Malakit study [13,14]), and secondary objectives were included to assess sexual behavior, use of addictive substances–which can modify sexual behavior–[15], and STI prevalence.

The aim of the present article is to describe the frequency of these risk behaviors, the prevalence of STIs, and the factors associated with condom use or transactional sex in order to guide potential interventions.

## Material and methods

### Study design

The study was a multicenter observational cross-sectional study designed to assess the efficacy of a new strategy to control malaria in illegal mines in FG (Malakit strategy) [13,14]. Capitalizing on the logistics and human resources involved (this population being difficult to access due to their illegal status and activity and their living deep in the forest), questions were added to study risk behaviors (sexual behavior and addictive substance use), and a blood sample was taken to test for STIs.

### Setting

Inclusions took place at rest sites (places where gold miners go to rest, buy supplies, or seek care) located along the two border rivers: the Maroni, at the border with Suriname (carried out by the French West Indies-French Guiana Center for Clinical Investigation–CIC Inserm 1424 –, Cayenne Hospital, FG) and the Oiapoque, at the border with Brazil (carried out by the Oswaldo Cruz Foundation, Brazil) [16].

### Participants

Inclusion criteria were: age 18 years or older, willingness to participate, working at an illegal gold mine in FG, having left the mine less than seven days before. Sampling was carried out by the snowball method. The number of participants was estimated to evaluate the efficacy of a new strategy to control malaria in this population (improvement in appropriate behaviors for malaria symptoms by 10 points, baseline = 0.54, alpha = 0.05, power 0.9), thus a sample size of 380 participants [14].

### Data collected

Questions were asked using a face-to-face questionnaire administered by a physician to assess sexual behavior and addictive substance use: socio-demographic data and gold mining activity, number of partners during the last year; condom use at last sexual intercourse; whether last sexual intercourse was commercial; cost of last condom used; past history of HIV testing; tobacco, alcohol, and drug consumption. The questionnaire was based on similar questionnaires used in the context of gold mining [2,17] and on validated questionnaires from reference health institutions [18–21]. The S1 Dataset presents the questionnaire in English and Portuguese (the language used with the participants), the origin of the questions, the data collected, definitions, and the modalities of the variables used in the analyses. The questionnaire had been previously field tested to assess comprehension of the questions in Portuguese and their acceptability.

A blood sample was only collected for participants included on the Maroni river (for funding and logistical reasons), and sent to Montpellier University Hospital (France) for serology testing: 1) Elisa–and Western Blot if positive–for HIV; 2) Hepatitis C (HCV) antibodies; 3) Hepatitis B (HBV) HBs antigen; 4) Syphilis, using the alternative algorithm initiated by treponemal screening, and, if positive, a non-treponemal test. All assays were done in accordance with the manufacturer’s instructions. HIV p24/antibodies, HBsAg, anti-HCV antibodies, and anti-treponema antibodies were assessed using fully automated chemiluminescence immunoassays (Alinity i, Abbott, IL, USA). The non-treponemal test, RPR Charbon, was performed on anti-treponema samples testing positive (Cypress Diagnostics, Belgium). Samples that tested positive for HIV were confirmed using the Geenius™ HIV1/2 Confirmatory Assay (Geenius, Bio-Rad, Marne la Coquette, France). The results were given to participants during a consultation at the primary care center in FG to allow for treatment of individuals who tested positive.

### Definitions

Multiple sexual partnerships were defined as having two or more sexual partners during the last year [19,21]. Excessive alcohol consumption was defined as having a score greater than or equal to 4 for men and 3 for women [18] (see S1 Dataset).

### Statistical methods

Data were analyzed using standard tests with a p-value<0.05. Univariate and multivariate analysis were carried out with Stata13 software. Multiple logistic regression models included variables with a p-value<0.2 in univariate analysis. The prevalence of STIs in 2015 and 2019 was calculated and presented graphically as a histogram with a 95% confidence interval. The design, setting, inclusion criteria, data collection procedure, and biological examinations (quantity of blood samples, methods, and testing laboratory) were identical in the 2015 and 2019 cross-sectional studies so that results could be compared [8].

### Ethics

A written informed consent form was collected for each participant. Since recruitment took place on the Surinamese and Brazilian borders, authorizations were obtained from their respective ethics committees (The CMWO–Commissie voor Mensgebonden Wetenschappelijk Onderzoek–in Suriname: N°DVG-738; the Fiocruz Ethics Committee in Brazil: N°2.560.415). The authorization to import human biological samples to FG was obtained from the French Ministry of Education and Research, process N° IE-2019-1099. The database was anonymized and declared to the French Data Protection Authority, CNIL.

## Results

### Study population

Between October and December 2019, 499 participants were included: 119 on the Oiapoque river and 380 on the Maroni river. The majority (96.6%) were born in Brazil, the median age was 39 years [Interquartile range (IQR) = 30–48], and the sex ratio M/F was 3 (Table 1). Women were younger and had a higher education level (Table 2). The participants had worked in gold mines for a median of 10 years and had left the forest a median of three times during the last year.

**Table 1:** Description of the cross-sectional study population, October-December 2019.

			n (%)
**Socio-demographic data**			
* *	**Age** **n = 497**	Median age [IQR]	39 [30–48]
		< = 29 yo	113 (22.7)
		30–44 yo	213(42.7)
		> = 45 yo	171 (34.4)
* *	**Sex** **n = 499**	Women	124 (24.8)
		Men	375 (75.2)
* *	**Education level** **n = 498**	None or primary	214 (43.0)
		Secondary	273 (54.8)
		Superior	11 (2.2)
* *	**Country of birth** **n = 499**	Other than Brazil	17 (3.4)
		Brazil	482 (96.6)
* *	**Inclusion site** **n = 499**	Maroni river	380 (76.2)
		Oiapoque river	119 (23.8)
**Gold mining activity**			
* *	**Time spent in gold mining** **n = 498**	≤ 5 years	172 (34.5)
		6–10 years	109 (21.8)
		11–15 years	66 (13.3)
		> 15 years	151 (30.4)
* *	**Time spent in the last mining site—n = 493**	Median time in years [IQR]	0.4 [0.2–1.3]
* *	**Travel time to the gold mining site** **n = 492**	<2 hours	31 (6.3)
		2 hours to half day	125 (25.4)
		1 day	121 (24.6)
		> 1 day	176 (35.8)
		Do not know	39 (7.9)
* *	**Type of activity** **n = 499**	Non-mobile activity	401 (80.4)
		Mobile activity	95 (19.0)
		Do not know	3 (0.6)
* *	**Number of mines they have worked at in the last 3 years** **n = 485**	< = 3	365 (73.2)
		>3	134 (26.8)
* *	**Number of trips away from mining site > 3 days during the last year** **n = 489**	Median [IQR]	3 [1–6]
**Sexual behavior**			
*** ***	**Number of sexual partners during the last year** **n = 494**	Median [IQR]	1 [1–5]
*** ***		0 partner	19 (3.8)
*** ***		1 partner	241 (48.8)
		2 à 5 partners	120 (24.3)
		> = 6 partners	99 (20.0)
*** ***		do not know/ do not want to answer	15 (3.0)
*** ***	**Condom use during the last sexual intercourse** **n = 499**	No	206 (41.3)
		Yes	279 (55.9)
		Do not want to answer	15 (2.8)
*** ***	**Condom bought (if used during the last intercourse)** **n = 269**	No	85 (31.6)
		Yes	176 (65.4)
		Do not want to answer	8 (3.0)
*** ***	**Condom price** **n = 117**	Median price in euros [IQR]	1 [0.7–1.7]
		Min-max	0.1–40
* *	**Transaction for the last sexual intercourse** **n = 499**	Payment given	111 (22.2)
		Payment received	11 (2.2)
		No transaction	331 (66.3)
		Do not want to answer	30 (6)
		Missing data/ do not know	16 (3.2)
* *	**Previous HIV test** **n = 499**	No	170 (34.1)
		Yes	320 (64.1)
		Do not know	5 (1.0)
		Do not want to answer	3 (0.6)
		Missing data	1 (0.2)
* *	**Date of the last HIV test** **n = 315**	within the past 12 months	116 (36.8)
		12 to 24 months ago	97 (30.8)
		more than 24 months ago	102 (32.4)
* *	**Result of the last HIV test (*N* = 320)**	Positive	0 (0)
		Negative	313 (97.8)
		Do not know	7 (2.2)
**Addictive substance use**			
* *	**Tobacco use** **n = 493**	No	290 (58.8)
		Yes	203 (41.2)
* *	**Excessive alcohol consumption** **n = 480**	No	205 (42.7)
		Yes	275 (57.3)
* *	**Crack or cocaïne consumption** **n = 488**	No	482 (98.8)
		Yes	6 (1.2)

**Table 2:** Sexual and addictive behavior according to gender (N = 499).

			Women N = 124 n (%)	Men N = 375 n(%)	p*
**Socio-demographic data**			** **	** **	** **
* *	**Age** **n = 497**	< = 29 yo	27 (23,9)	86 (76,1)	**0,020** **
		30–44 yo	65 (30,5)	148 (69,5)	
		> = 45 yo	31 (18,1)	140 (81,9)	
	**Sex** **n = 498**	Women	32 (14,9)	182 (85,1)	**<0,001**
		Men	92 (32,4)	192 (67,6)	
	**Education level** **n = 499**	None or primary	9 (52,9)	8 (47,1)	**0,006**
		Secondary or superior	115 (23,9)	367 (76,1)	
	**Country of birth** **n = 499**	Other than Brazil	102 (26,8)	278 (73,2)	0,066
		Brazil	22 (18,5)	97 (81,5)	
**Sexual behavior**			** **	** **	** **
* *	**Number of sexual partners during the last year** **n = 479**	0 or 1 partner	97 (37,3]	163 (62,7)	**<0,001**
		2 to 5 partners	19 (15,8)	101 (84,2)	
		> = 6 partners	4 (4,0)	95 (96,0)	
	**Condom use during the last sexual intercourse** **n = 485**	No	78 (37,9)	128 (62,1)	**<0,001**
		Yes	43 (15,4)	236 (84,6)	
	**Condom bought (if used during last sexual itnercourse** **n = 279**	No	13 (14,8)	75 (85,2)	0,127
		Yes	27 (14,8)	155 (85,2)	
		Do not know/ do not want to answer	2 (22,2)	7 (77,8)	
	**Previous HIV test** **n = 499**	No	10 (5,9)	160 (94,1)	**<0,001**
		Yes	113 (35,3)	207 (64,7)	
	**Date of the last HIV test** **n = 315**	within the past 12 months	49 (42,2)	67 (57,8)	**0,014**
		12 to 24 months ago	39 (40,2)	58 (59,8)	
		more than 24 months ago	25 (24,5)	77 (75,5)	
	**Transaction for last sexual intercourse** **n = 480**	Payment given	0 (0)	111 (100)	**<0,001**
		Payment received	10 (90,9)	1 (9,1)	
		No transaction	109 (32,9)	222 (67,1)	
		Do not want to answer	2 (6,7)	28 (93,3)	
		Missing data/do not know	3 (18,7)	13 (81,3)	
	**Having a STI**	No	82 (25.8)	236 (74.2)	**0.293**
		Yes	20 (32.3)	42 (67.7)	
**Addictive substances use**					
* *	**Tobacco use** **n = 493**	No	76 (26,2)	214 (73,8)	0,248
		Yes	44 (21,7)	159 (78,3)	
	**Excessive alcohol consumption** **n = 480**	No	54 (26.3)	151 (73.7)	0.440
		Yes	64 (23.3)	211 (76.7)	
	**Crack or cocaïne consumption** **n = 488**	No	121 (25,1)	361 (74,9)	0,533
		Yes	1 (16,7)	5 (83,3)	

* standard tests (chi2, fischer, student).

** in bold: Significant variables.

### Sexual behavior

- **Number of partners during the last year.** Half of the participants (52.6%) declared having had a single or no partner during the last 12 months. Among the 44.3% (n = 219/499) with multiple sexual partnerships, the median was five different partners during the last year [IQR = 3–10], a phenomenon that was greater among men than women (Table 2). Women declaring sex work declared a median of 20 partners during the last year [10–50], while for other women the median was one [1–4].
- **Condom use.** Among the 279 (55.9%) participants who declared having used a condom during the last sexual intercourse, 65.2% had bought it. The condoms were paid for in euros (37.4%), reals (31.0%), Surinamese dollars (9.1%), grams of gold (8.6%), or other (13.9%). The median price in euro-equivalent was one euro for a condom (min-max = 0.06 to 40 euros). The price was higher when the condom was paid in grams of gold—so probably directly on the gold mining sites (median equivalent of 13 euros [10–13]) than in euros (median of 1 euro [0.8–1.6]), reals (median equivalent of 0.8 euros [0.3–1.1]), or Surinamese dollars (median equivalent of 0.7 euros [0.7–1.2]) (p<0.001)–more probably bought on rest sites.

After multivariate analysis, condom use was associated with being a man, (AOR = 1.8 [1.1–3.0]), having multiple sexual partners (AOR = 2.6 [95% CI = 1.6–4.1]), and transactional sex (AOR = 12.3 [5.3–28.3]) (Table 3).

**Table 3:** Factors associated with condom use during the last sexual intercourse (N = 485).

			No condom use N = 206	Condom use N = 279	Univariate analysis		Multivariate analysis^c^
					OR^a^ [95%CI]	p	AOR^b^ [95%CI]
**Socio-demographic data**							** **
* *	**Age** **n = 483**	< = 29 yo	38 (34.2)	73 (65.8)	1	**0.019** ^**d**^	1
		30–44 yo	103 (49.5)	105 (50.5)	0.5 [0.3–0.9]		0.5 [0.3–0.9]
		> = 45 yo	65 (39.6)	99 (60.4)	0.8 [0.5–1.3]		0.5 [0.3–1.0]
* *	**Sex** **n = 485**	Women	78 (64.5)	43 (35.5)	1	**<0.001**	1
		Men	128 (35.2)	236 (64.8)	3.3 [2.2–5.1]		1.8 [1.1–3.0]
* *	**Education level** **n = 485**	None or primary	81 (39.5)	124 (60.5)	1	0.259	
		Secondary or superior	125 (44.6)	155 (55.4)	0.8 [0.6–1.2]		
* *	**Country of birth** **n = 485**	Other than Brazil	11 (68.7)	5 (31.3)	1	**0.031**	
		Brazil	195 (41.6)	274 (58.4)	3.0 [1.1–9]		
**Gold mining activity**							** **
* *	**Time spent in gold mining** **n = 484**	< = 10 years	116 (42.6)	156 (57.4)	1	0.966	
		>10 years	90 (42.5)	122 (57.5)	1.0 [1.0–1.0]		
* *	**Time spent in the last mining site** **n = 479**	< = 6 months	115 (41.5)	162 (58.5)	1	0.507	
		> 6 months	90 (44.6)	112 (55.4)	1.0 [1.0–1.0]		
* *	**Travel time to the gold mining site** **n = 478**	< = 1 day	72 (46.5)	83 (53.5)	1	0.437	
		> 1 day	114 (40.1)	170 (59.9)	1.3 [0.9–1.9]		
		do not know	17 (43.6)	22 (56.4)	1.1 [0.6–2.3]		
* *	**Type of activity** **n = 482**	Non-mobile activity	165 (42.3)	225 (57.7)	1	0.694	
		Mobile activity	41 (44.6)	51 (55.4)	0.9 [0.6–1.4]		
* *	**Number of mines worked at in the last 3 years** **n = 485**	< = 3	154 (43.8)	198 (56.2)	1	0.355	
		>3	52 (39.1)	81 (60.9)	1.0 [1.0–1.0]		
* *	**Number of trips away from mining site > 3 days during the last year** **n = 476**	< = 4	134 (42.3)	183 (57.7)	1	0.715	
		>4	70 (44.0)	89 (56.0)	1.0 [1.0–1.0]		
**Sexual behavior**							
* *	**Number of sexual partners during the last year** **n = 472**	0 or 1 partner	155 (61.3)	98 (38.7)	1	**<0.001**	1
		More than 1 partner	46 (21)	173 (79)	5.9 [3.9–9.0]		2.4 [1.5–4.0]
* *	**Transaction for last sexual intercourse** **n = 485**	No transaction	191 (57.9)	139 (42.1)	1	**<0.001**	1
		Payment given or received	8 (6.6)	114 (93.4)	19.6 [9.3–41.4]		12.6 [5.4–29.2]
		Do not want to answer/missing data	7 (21.2)	26 (78.8)	5.1 [2.1–12.1]		3.3 [1.2–9.3]
* *	**Previous HIV test** **n = 485**	No	63 (37.7)	104 (62.3)	1	**0.124**	
		Yes	140 (45.0)	171 (55.0)	0.7 [0.5–1.1]		
**Addictive substances use**						** **	
* *	**Tobacco use** **n = 479**	No	130 (45.6)	155 (54.4)	1	**0.064**	
		Yes	72 (37.1)	122 (62.9)	1.4 [1–2.1]		
* *	**Excessive alcohol consumption** **n = 485**	No	96 (47.8)	105 (52.2)	1	**0,037**	
		Yes	101 (38.1)	164 (61.9)	1.5 [1.0–2.2]		
* *	**Crack or cocaine consumption** **n = 476**	No	200 (42.5)	271 (57.5)	1	0.364	
		Yes	3 (60)	2 (40)	0.5 [0.08–3.0]		

a Odds-ratio.

b Adjusted Odds-Ratio after logistic regression.

c Goodness-of-fit of the model (Hosmer-Lemeshow) = 0.832.

d in bold: Variables included in the multivariate analysis.

- **Transactional sex.** The last sexual intercourse was transactional for 24.4% of the population: 22.2% had given compensation and 2.2% were paid (Table 1). Among the participants who declared having received compensation, there were 10 women and one man (Table 2). Two of the women had indicated sex work as their primary or secondary activity at the time of the interview. The others were: bar owner (2), housekeeper/cook (2), traveling saleswoman (2), other (2). Among the three women declaring sex work, the last intercourse was transactional sex for two of them.

Transactional sex was associated with condom use (AOR = 10.5 [4.7–23.4]) and having more than one sexual partner during the last year (AOR = 7.5 [4.0–14.1]), (Table 4). For the last transactional sexual intercourse, condoms were used by 100% of women and 92.9% of men (p = 0.382).

**Table 4:** Factors associated with transactional sex (univariate and multivariate analysis) (N = 453).

			No transaction N = 331 n (%)	Transaction N = 122 n (%)	Univariate analysis		Multivariate analysis^c^
					OR^a^ [95%CI]	p	AOR^b^ [95%CI]
**Socio-demographic data**							** **
* *	**Age** **n = 451**	< = 29 yo	78 (78.8)	21 (21.2)	1	**<0.001** ^**d**^	1
		30–44 yo	154 (79.4)	40 (20.6)	1 [0.5–1.7]		1.3 [0.6–2.8]
		> = 45 yo	98 (62.0)	60 (40.0)	2.3 [1.3–4.1]		3.3 [1.5–7.0]
	**Sex** **n = 453**	Women	109 (91.6)	10 (8.4)	1	**<0.001**	1
		Men	222 (66.5)	112 (33.5)	5.5 [2.8–10.9]		2.5 [1.0–6,4]
	**Education level** **n = 453**	None or primary	121 (65.4)	64 (34.6)	1	**0.002**	
		Secondary or superior	210 (78.4)	58 (21.6)	0.5 [0.3–0.8]		
	**Country of birth** **n = 453**	Other than Brazil	14 (87.5)	2 (12.5)	1	**0.153**	
		Brazil	317 (72.5)	120 (27.5)	2.6 [0.6–11.8]		
	**Inclusion site^e^** **n = 453**	Maroni river	267 (70.8)	110 (29.2)	1	**0.012**	
		Oiapoque river	64 (84.2)	12 (15.8)	0.5 [0.2–0.9]		
**Gold mining activity**							** **
* *	**Time spent in gold mining** **n = 452**	< = 10 years	193 (76.3)	60 (23.7)	1	**0.098**	
		>10 years	138 (69.4)	61 (30.6)	1 [1–1]		
	**Time spent at the last mining site** **n = 447**	< = 6 months	194 (72.9)	72 (27.1)	1	0.898	
		> 6 months	133 (73.5)	48 (26.5)	1.0 [1.0–1.0]		
	**Travel time to the gold mining site** **n = 446**	< = 1 day	102 (67.1)	50 (32.9)	1	**0.107**	
		> 1 day	196 (76.6)	60 (23.4)	0.6 [0.4–1]		
		do not know	29 (76.3)	9 (23.7)	0.6 [0.3–0.7]		
	**Type of activity** **n = 450**	Non-mobile activity	264 (71.9)	103 (28.1)	1	**0.149**	
		Mobile activity	66 (79.5)	17 (20.5)	0.7 [0.4–1.2]		
	**Number of mines worked at in the last 3 years** **n = 453**	< = 3	244 (74.4)	84 (25.6)	1	0.308	
		>3	87 (69.6)	38 (30.4)	1.0 [1.0–1.0]		
	**Number of trips away from mining site > 3 days during the last year** **n = 445**	< = 4	217 (73.3)	79 (26.7)	1	0.907	
		>4	110 (73.8)	39 (26.2)	1.0 [1.0–1.0]		
**Sexual behavior**							
* *	**Number of sexual partners during the last year- n = 440**	0 or 1 partner	225 (92.6)	18 (7.4)	1	**<0.001**	1
		More than 1 partner	97 (49.2)	100 (50.8)	12.9 [7.4–22.5]		7.5 [4.0–14.1]
	**Condom use during the last sexual intercourse—n = 452**	No	191 (96.0)	8 (4.0)	1	**<0.001**	1
		Yes	139 (54.9)	114 (45.1)	19.6 [9.3–41.4]		10.5 [4.7–23.4]
	**Previous HIV test** **n = 447**	No	107 (72.3)	41 (27.7)	1	0.66	
		Yes	222 (74.3)	77 (25.7)	0.9 [0.6–1.4]		
	**Having a STI** **n = 377**	No	224 (70.9)	92 (29.1)	1	0.95	
		Yes	43 (70.5)	18 (29.5)	1.0 [0.6–1.8]		
**Addictive substances use**							
* *	**Tobacco use** **n = 449**	No	204 (75.3)	67 (24.7)	1	**0.151**	
		Yes	123 (69.1)	55 (30.9)	1.3 [0.9–2.1]		
	**Excessive alcohol consumption** **n = 453**	No	150 (76.1)	47 (23.9)	1	**0.085**	
		Yes	165 (68.8)	75 (31.2)	1.5 [0.9–2.2]		
	**Crack or cocaine consumption** **n = 447**	No	323 (73.1)	119 (26.9)	1	0.411	
		Yes	3 (60.0)	2 (40.0)	1.8 [0.3–11.0]		

a Odds-ratio.

b Adjusted Odds-Ratio after logistic regression.

c Goodness-of-fit of the model (Hosmer-Lemeshow) = 0.940.

d in bold: Variables included in the multivariate analysis.

e This difference between persons included on the Maroni river and on the Oiapoque river maybe explained by a difference in the way the respective teams on either borders asked the related questions since 24.4% of the respondents did not want to answer on the Oiapoque river compared with only 0.2% on the Maroni river.

### Sexually transmitted infections

**Fig 1:**
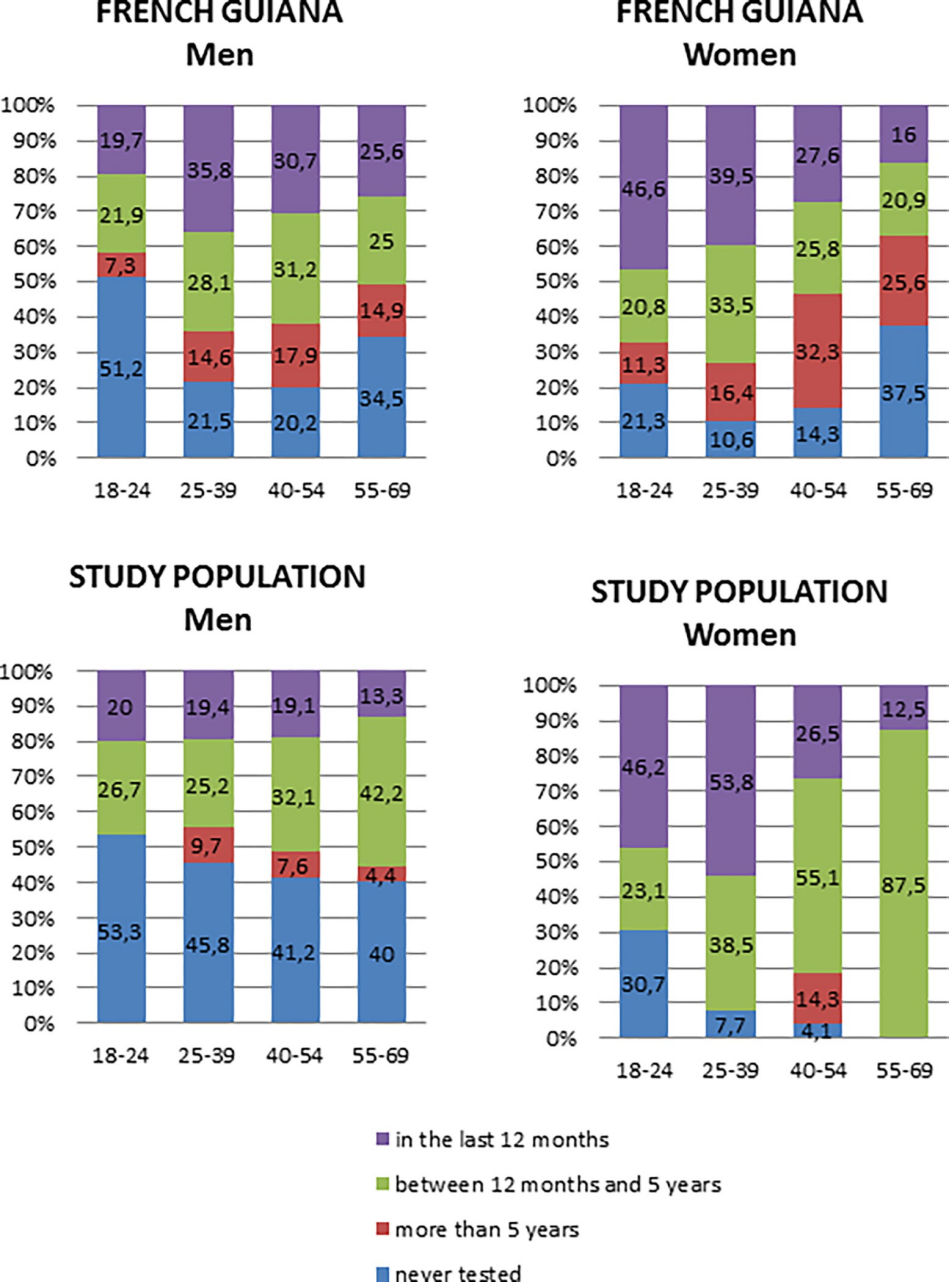
Previous HIV test among the study population and the French Guianese population by sex and age.

**Fig 2:**
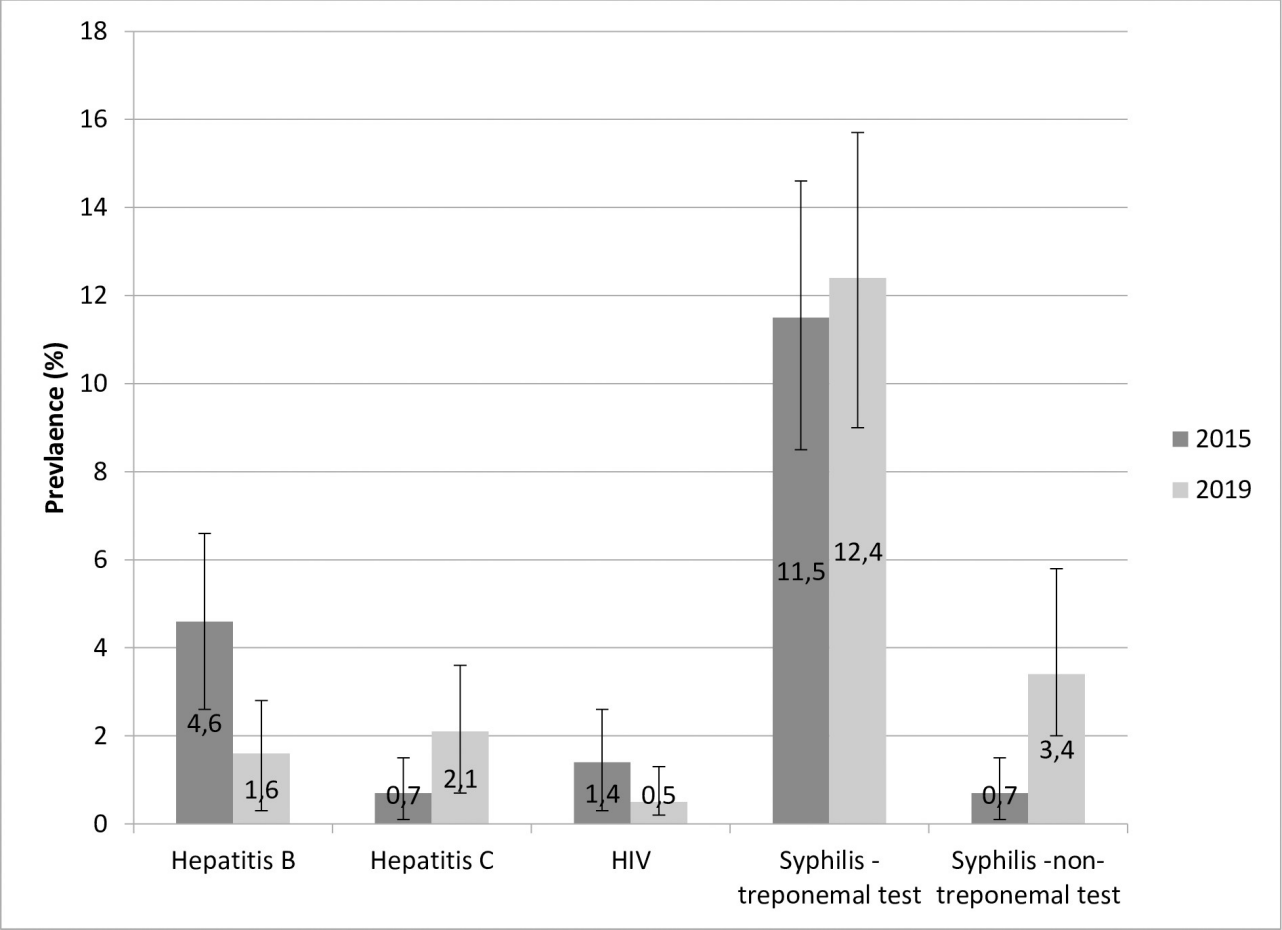
Prevalence of HBC, HCV, HIV, and syphilis among illegal gold miners working in French Guiana in 2015 and 2019.

- **HIV testing.** Three hundred and twenty participants declared having had a previous HIV test (64.1%): 36.8% during the past 12 months, 30.8% 12 to 24 months ago, and 33.2% more than 24 months ago. A greater proportion of women had been previously tested than men (91.1% versus 55.2%) (Fig 1). Of the 320 participants who had had an HIV test, 313 (97.8%) said the test was negative, and seven did not know the result (2.2%).

- **Prevalence of STIs.** Among the 380 participants included on the Maroni river, two tested positive for HIV at inclusion (prevalence = 0.5% (95% CI: 0.1–2.1) (Fig 2). The first was a 24-year-old man who had worked for two years as a gold miner. He had had one sexual partner in the previous year and did not use a condom during the last sexual intercourse. He reported a negative HIV test in 2016. He also had a positive treponemal serology. The second was a 45-year-old man who had worked in gold mining for 19 years. He had had three sexual partners within the year, the last sexual intercourse was paid, and he used a purchased condom. He declared having had a negative HIV test in 2018.

Eight participants–all men–had a positive HCV serology, thus a prevalence of 2.1% (95% CI: 0.7–3.6). Among these, six had low antibody titers. Six participants had positive HBs antigen (1.6% (95% CI: 0.3–2.8)), all men as well. The treponemal test was positive for 12.4% (95% CI: 9.0–15.7) of participants. Among those who tested positive, 27.7% had a positive non-treponemal test, which represented 3.4% of the included population (95% CI: 2.0–5.8). Positive treponemal tests were significantly more frequent among women (19.6% versus 9.7% among men, p = 0.009) and among older people (1.4% among 18–29 yo, 13.0% among 30–44 yo, 17.9% among > 45 yo, p = 0.003). The same observations were made with non-treponemal tests. No HIV/HCV/HBV co-infection was found in the study population nor any co-infection with syphilis, except for the participant with positive HIV and treponemal serology.

In total, 62 participants had at least one STI (HIV, HCV, HBsAg, treponemal serology), representing 16.3% of the study population (95% CI: 12.6–20.0). The only factor associated with having an STI was being older. None of the women reporting sexual activity had STIs.

### Addictive substance use

- **Tobacco.** In the study population, 41.1% smoked tobacco, with no difference between age groups or genders (36.7% of women, 42.6% of men, p = 0.248). The median age of first smoking was 17 [IQR = 14–20]. The median quantification of cigarette consumption was 15 pack-years [IQR = 5.5–30].
- **Alcohol consumption.** Twenty-six percent of the surveyed participants declared never drinking alcohol, 38.7% less than once a month, 21.0% two to four times a month, and 13.2% more than two times a week (N = 380: This question was asked only on the Maroni river). Among the overall study population (N = 499), when they drank alcohol, 14.7% had one or two drinks, 14.9% had three to four drinks, 13.8% had five to nine drinks, and 56.5% had more than ten drinks of alcohol. To the question “how often do you have more than six standard drinks?”, 41.1% of respondents answered never, 35.7% once a month or less, 17.6% once a week, and 5.6% almost every day. According to the WHO classification [18], 57,3% of the study population had an excessive alcohol consumption, with no difference between men and women (58.3% versus 54.2% respectively, p = 0. 440). Tobacco use and alcohol consumption were linked. Regarding sexual behavior, excessive alcohol consumers had more sexual partners (25.2% had more than five partners during the last year versus 15.7%, p = 0.017) and reported using condoms more frequently (61.9% versus 52.2%, p = 0.037).
- **Drugs.** Forty-three participants (43/499, 8.6%) declared consuming marijuana, occasionally (30.2%), weekly (32.6%), or daily (37.2%). Six people (1.2%) reported consuming crack and/or cocaine, occasionally or weekly. They were mainly men (5/6), aged from 24 to 44, and had a secondary school education level (4/5, 1 missing data). Two had had previous transactional intercourse (2/5, 1 missing data), one of whom did not use a condom. No other drug use was declared.

## Discussion

This study documents for the first time the risk behaviors of illegal gold miners regarding sex and addictive substance consumption in FG. The study shows that transactional sex was very prevalent but that condom use was frequent. One third of the men in our study reported paying for their last sexual intercourse. This proportion was much higher than the 8.6% on the Maroni river, 4.6% in coastal French Guiana, and 1.9% in continental France of men reporting commercial sex during the last year [22–24]. The estimate among precarious urban migrants in FG (among whom 25% were from Brazil) was 15% in the past 12 months [25]. While the frequent use of transactional sex among men was expected, the relatively low proportion of women declaring sex work was an unexpected finding. Although we may have underestimated this stigmatized activity, these results are a far cry from the stereotype that depicts the activity of the majority of women at gold mines as consisting of sex work. Nevertheless, transactional sex among women is still much higher than in the French Antilles (0.1% of women having being paid in the last 12 months) or in continental France (0.01%), but close to the 8.2% of women who declared having received money or drugs in exchange for sex in a population of precarious urban migrants in FG [25].

Women had fewer sexual partners–except for those declaring sex work–, had fewer STIs, and were more frequently tested for HIV than men. Prevalence of STIs seemed greater than in the general French Guianese population, especially for syphilis.

### Risk behaviors for STIs

Men used condoms more often than women, for transactional or single partner sex, as described elsewhere [24]. For transactional sex, a potentially high risk activity, most surveyed men declared having used a condom, proportion that is comparable to those observed in other surveys in French Guiana in the general population and in urban migrants [24,25]. The systematic use of condoms by women during transactional sex with clients was similar to that of sex workers in French Guiana [21], but much higher than in Brazil where only 80.5% of sex workers systematically used condoms [26]. Thus, despite their very high price in the forest, condoms appear to be easily accessible and commonly used in transactional sex.

Although the observed HIV prevalence did not seem alarming, the high proportion of Brazilians among newly-diagnosed patients seen in primary care centers warrants the surveillance of HIV prevalence in this population [2,9]. In this study, 64% of the participants had been previously tested for HIV, which was a lower percentage than in the general population in FG (78.1%), but quite close to the proportion in continental France (61.7%) [21]. More women had been tested than men, which is common and partly due to testing during pregnancy.

Replicating the 2015 survey findings, the prevalence of syphilis remained high, with an increase of non-treponemal test positivity suggesting recent infection. The overall prevalence of positive treponemal tests was comparable to the HIV hospital cohort (13.2%), among whom the prevalence of positive non-treponemal tests was 11%–substantially less than the 27.7% among the surveyed gold miners (M. Nacher, personal communication). Although a positive treponemal serology does not distinguish syphilis from yaws or pinta, these diseases have not been observed in French Guiana for decades. This high prevalence of active syphilis presumably reflects lack of care for a non-painful disease, particularly among women, who may have cervical lesions–which are not visible and not painful–and who may be less inclined to consult than when having symptomatic STIs. Hepatitis C prevalence was low and HBsAg prevalence was lower than that observed in 2015, but seemed greater than in the Americas and similar to FG or the Brazilian state of Maranhão where the majority of gold miners come from [2,27].

Excessive alcohol consumption and tobacco use were very frequent, but, contrary to our expectations, cocaine or crack consumption did not appear to be widespread. Over half of the study population reported an excessive consumption of alcohol. This is very high considering that site managers often prohibit drinking for safety and productivity reasons [11]. There are few studies on the subject but a study at an illegal mining site in the state of Pará (Brazil) showed that 81% of participants routinely ingested alcoholic beverages [28]. The proportion of smokers was much higher than in Brazil, respectively 19.3% and 11.4% [29]. Except for cannabis and very rarely cocaine or crack, no other drugs were reported. The associated stigma may have led some participants to not report their drug use, but the results suggest a much rarer use than common presuppositions attribute to gold miners: the belief that miners consumed large quantities of drugs to cope with difficult working conditions was hence refuted in this study [30].

The present study has a number of limitations: since it was designed to assess malaria prevalence, which was expected to be high, confidence intervals for STIs are wide and estimates are thus difficult to interpret; even though the questionnaire was administered by a medical doctor at an isolated site, some responses may be biased by under-reporting due to stigma linked to sex work or drug use and/or by the desire to give the socially desirable answer.

However, despite these limitations, these results show some marked trends that are important to optimize the fight against STIs and HIV. Although the findings are far from commonly-held extreme representations about gold miners, they reveal behaviors conducive to the spread of STIs, with multiple sexual partnerships, frequent transactional sex, and remoteness from the health care system making them vulnerable populations. Furthermore, there is regular contact between gold miners and the local population, when the miners come to rest sites (cross-border areas where they go to rest, sell their gold, get supplies, or visit their families [2]) or through sex workers who live on the rivers and regularly go to work at gold mining sites. The ultimate integration of sexual networks through bridging populations reemphasizes the need for interventions to target both vulnerable groups and the general population [23,31]. Access to condoms must be facilitated to maintain a high rate of use during commercial sex, but also to increase use in the context of multiple sexual partnerships. Efforts should focus on increasing condom distribution in border towns, primarily to FSWs. STI screening must also be reinforced, preferably using rapid tests, such as those for HIV and syphilis, due to the high mobility of these populations, who do not necessarily come to get their results [32,33]. Health facilities located along the borders are suitable places for comprehensive prevention and testing. Beyond the sensitization of the health professionals working in these areas, rapid tests should be made widely available to these populations.

## Conclusion

Our initial hypothesis was that gold miners had many sexual risk factors and addictions. Our findings generally confirm this, but with some unexpected nuances: men working in illegal gold mining in French Guiana very frequently resorted to transactional sex, but the majority of women at mining sites did not engage in sex work. Sexually transmitted infections, especially syphilis, seemed more prevalent in this population. Condom use was inconsistent, but for transactional sex it was high. Excessive alcohol consumption was very frequent, but, contrary to our beliefs, crack/cocaine use was rare. Access to STI screening and condoms must be facilitated in order to prevent the spread of STIs.

## Supporting information

S1 Dataset Questionnaire used in the 2019 study in English and Portuguese (language used with the participants), origin of the questions, data collected, definitions, and modalities of the variables used in the analyses. (XLSX)
